# Supplementary material for: Nutritional and meiotic induction of transiently heritable stress resistant states in budding yeast
Source: Microb Cell. 2018 Oct 29;5(11):511–21. doi: 10.15698/mic2018.11.657 (PMC6244294; doi:10.15698/mic2018.11.657)
Supplement: Supplementary file 1 [file mic-05-511-s01.pdf]

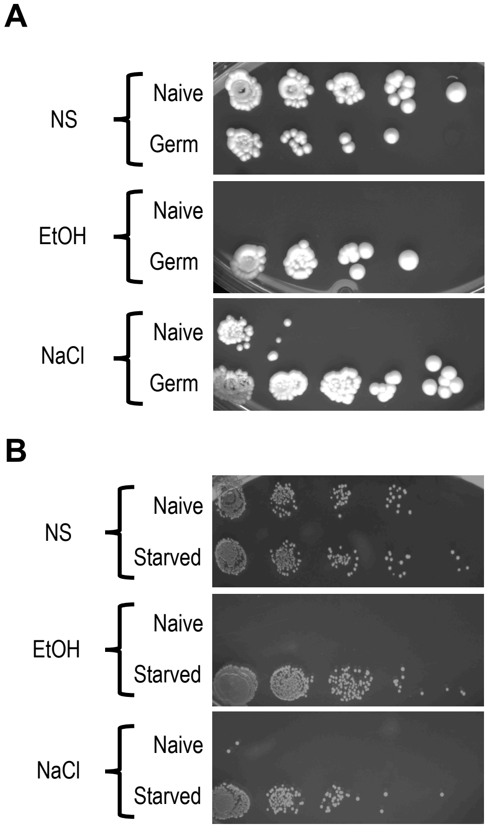

Figure S1. Germination and glucose starvation induce cross-stress resistance to ethanol and NaCl. A) 10-fold serially diluted spot assay of germinating cells treated with ethanol (EtOH) or NaCl. B) 10-fold serially diluted spot assay of 12 hour glucose starved cells treated with EtOH or NaCl.
